# From Inpatient Notes to Outpatient Followup: Enhancing the Rhinology Service in a Tertiary Centre through Student Led Projects

**DOI:** 10.1155/2015/197823

**Published:** 2015-08-06

**Authors:** M. Sayma, R. Hyne, M. Sharma, L. Kyle, M. Abo Khatwa, I. MacKay-Davies, A. Poulios, H. S. Khalil

**Affiliations:** Plymouth Hospitals NHS Trust and Peninsula College of Medicine and Dentistry, Plymouth, UK

## Abstract

*Introduction*. Medical students can use systems to help improve the quality of care in a unit. Following the review of care within the ENT department at a tertiary centre a number of quality improvement projects were put in place. *Methods*. The following interventions were established: (1) creation of an outpatient telephone enquiry clinic, (2) development of a rhinology database, (3) introduction of operative note templates, and (4) construction of electronic discharge summary templates (eDSTs). *Discussion and Outcomes*. (1) Consultant telephone inquiry clinics were successfully organised and showed high levels of patient satisfaction. (2) A database to collect patient reported outcome measures was piloted within rhinology outpatients; the results suggest that such a database would be simple to introduce and yield benefits for patients and the department. (3) Operative note templates for FESS procedures were implemented with a view to improving the continuity of care onto the ward; these have become well established and further steps to integrate these into routine care are being taken. (4) eDSTs specific to FESS and septorhinoplasty procedures were introduced with a view to increasing completion speed of templates and adherence to Royal College of Physician Guidance.

## 1. Introduction

Medical students have been shown to have the ability to improve patient care outcomes when given the opportunity to be involved in quality improvement in healthcare settings [1]. Senior medical students were offered the opportunity as part of a student selected component in rhinology to undertake a series of quality improvement project assessing the efficacy of a number of processes in a British rhinology unit. This series was selected to sequentially assess and improve points of patient care from recording operative data to reviewing these patients as outpatients.

### 1.1. Creation of an Outpatient Telephone Enquiry Clinic

With pressured clinical resources failing to accommodate the increasing backlog of follow-up appointments, telephone clinics have been proposed as a novel alternative of reducing the vast number of patients waiting for routine followup [2]. Traditionally, surgeons have reviewed postoperative patients face to face (FTF) as this postoperative outpatient review is important not only for patient reassurance but also for auditing the efficacy and complications of surgical procedures [3, 4]. However, reports performed in 2008/2009 and 2010/2011 stated that many follow-up appointments are unnecessary for patients undergoing routine surgery [2]. With “best practice” defined as having a “no wait culture,” the South West Strategic Health Authority (SW SHA) aims to improve the time in which care is delivered through safely and effectively substituting specific FTF follow-up appointments with telephone consultations [2].

### 1.2. Development of a Rhinology Database

Standardisation and comparison of surgical outcomes are important to ensure best patient care and are a requirement for revalidation. At present many departments report surgical outcomes via data collected from coding. Although this may not necessarily reflect patient reported outcomes, departments may have little other options. Patient-reported outcome measures (PROMs) are increasingly gaining acceptance as important and valid measures of symptoms, experiences, and quality of life. Patient communication, care, and outcomes have been shown to improve as a result of integrating their collection into routine clinical practice [5].

Since 2009, the BRS has provided an online tool for recording outcomes and evaluating performance against national averages. However, problems with access and a large number of obligatory fields have inhibited its use [6]. Therefore, this project aimed to pilot and evaluate an in-house, computerised database for collection of PROMs, introduced as a means of assessing the effect of interventions and aiding reporting of outcomes.

### 1.3. Operative Note Templates for FESS Procedures

Operative notes are the only comprehensive documented evidence of what happens in surgery [7]. They serve as a method of communication between theatre staff and ward staff. Accurate and detailed notes are important to provide satisfactory postoperative care and serve as proficient evidence in medicolegal situations [7–12]. The GMC states that good note keeping is an essential part of good medical care [7, 13], and the Royal College of Surgeons (RCS) says that medical records are “fundamental for clinical care and audit of surgical services” [14]. The RCS published guidelines on the basic components that all operative notes should include in order to communicate the necessary information and produce a medicolegally safe document [10, 14].

Problems arise with hand-written operative notes, such as legibility of the surgeons' handwriting [7]. Up to 11.4% of drug errors made on wards are due to illegible handwriting in operative notes [12]. Handwritten operative notes may not be complete; a template devised for use in kidney cancer showed an increase in completion rates from 68% in dictated notes to 92% in the online template [15].

Through introducing an operative note template for functional endoscopic sinus surgery (FESS) and nasal polypectomy procedures, we aim to improve the completeness of operative notes, create a safer communication pathway between surgical staff and ward based staff, and save time taken to fill out operative notes.

### 1.4. eDSTs Specific Production

Discharge summaries communicate essential clinical information from inpatient settings to primary care. Information transmission was previously conducted through dictated letters completed by administrative staff. This often resulted in poor quality information being given to primary care providers, in an untimely manner [16]. As a result GP's called for the introduction of “electronic discharge summaries” (eDSs) to increase quality and speed of information transfer [17].

The hospital involved in this project introduced an eDS system in 2008 and in 2010 the “Clinical Data Standards Assurance programme” began a project to deliver national, clinically assured eDSs [18]. Despite implementation of eDSs, problems have still arisen regarding their timeliness and content and these have been well discussed in the literature [19–21]. Further literature analysis highlighted that these issues may lead to deficits regarding patient safety and continuity of care [22]. The Royal College of Physicians recommends that a DS should be produced for every patient and should contain a set of key subheadings [23].

Recent evidence has suggested that the addition of “prompting systems” to electronic discharge summaries may improve their content quality, resulting in improved patient safety [24, 25].

For these reasons, the quality of electronic discharge summaries in Rhinology at Derriford Hospital was analysed, with a view to the introduction of a “prompting system” or custom eDS templates to improve their quality.

## 2. Methods

### 2.1. Creation of Outpatient Telephone Enquiry Clinic

In order to maximise the possibility of successfully contacting patients, whilst utilising clinician time efficiently five patients were selected using the inclusion and exclusion criteria. The inclusion criteria comprised of surgical procedures with low-risk complications such as functional endoscopic sinus surgery (FESS), diathermy of inferior turbinates (DITs), polypectomy, and septoplasty. Sinonasal tumours, endoscopic dacryocystorhinostomy (DCR), nasal biopsies, and septorhinoplasty were excluded due to the expected need for regular followup. During each consultation, patients were asked questions relevant to their procedure using either a Sino-Nasal Outcome Test (SNOT22) or Nasal Obstruction and Septoplasty Effectiveness (NOSE) scale and this was completed using the ENT electronic database. The time taken per consult was recorded and a mixed methodology approach, including telephone and FTF interviews, was used to obtain patient views on suitability of the TIC and monitor patient satisfaction.

### 2.2. Development of a Rhinology Database

A Microsoft access database was developed for use in rhinology outpatients. This database allows recording of patient ID, demographics, diagnosis, surgery performed, and date of surgery in addition to the appropriate PROM. Two validated PROMs were included: Sino-Nasal Outcome Test 22 for use in rhinosinusitis and Nasal Obstruction Symptom Evaluation for use in nasal obstruction.

This database was piloted in two settings: outpatients and a telephone follow-up clinic. In each clinic the database was completed for four patients and the time taken was recorded. In addition, an opinion on the database was sought from an ENT surgeon with expertise in database design.

### 2.3. Introduction of Operative Note Templates

A pilot study trialling the use of an operative note template for FESS and nasal polypectomy was conducted. The template was developed using the RCS operative note guidelines to ensure the 14 points in RCS guidelines were included. The template was piloted in theatre by four surgeons and then evaluated straight after using Likert scales. The operative notes entered the patient notes, where four recovery nurses evaluated them. Following evaluation from both groups, the template was adapted to appease their suggestions and include important information. The template was repiloted by the same four surgeons and recovery ward nurses to see if opinions and the usability of the template had improved, with the eventual aim of computerising the template. The inclusion criterion was any FESS, nasal polypectomy, and functional endoscopic nasal surgery (FENS) taking place. Exclusion criteria included any operations not stated above.

### 2.4. Construction of Electronic Discharge Summary Template

A prospective case note review was conducted of sequential patients who had undergone rhinology procedures. The entries on the generic eDSs to the GP were compared to the information in clinical records. Discrepancies were noted. The content of each eDSs was assessed against RCP generic record guidance to screen for omissions. Five clinicians were timed when completing eDSs to assess completion time. Following the audit, a custom eDS template was designed using lists of common symptoms, risks, and warnings (among other subheadings) prompting clinicians to complete all required data.

## 3. Discussion and Outcomes

### 3.1. Creation of Outpatient Telephone Enquiry Clinic

The TEC for postoperative rhinology patients appears to be a safe and cost-effective alternative to FTF followup, both acceptable to and appreciated by patients. The initial pilot study has shown that TECs can avoid unnecessary outpatient appointments and increase the availability of clinic slots by providing a quicker method of reviewing patients. Our experience suggests that future TECs should be led by a senior clinician to adequately address the complexity of questions asked and maintain patient safety. Patient views regarding the TEC proved promising, with patients stating the preference for telephone consultations as it reduced waiting and travel times and minimised the need to take time of work and the cost of hospital parking.

Overall, the proposed intervention is a safe and effective substitution of FTF consultations that provides efficient health care which is equitable and patient-centred, validating its future sustainability of the inclusion of TEC in routine follow-up care.

### 3.2. Development of a Rhinology Database

The pilot provided information about the utility of the database. The mean time taken to complete the database was 3.25 minutes in the outpatient clinic and 4 minutes in the telephone clinic. In addition the pilot, together with the opinion of our database expert, allowed a range of positive and negative aspects of the database to be identified.

Positive aspects included

- ease of use,
- time efficiency,
- PROM simple for patients to understand.

Negative aspects included

- not available on the network,
- no ability to delete entries from database if incorrectly entered,
- no easy access to database tables.

The results from this pilot suggest that a fit for purpose database would not greatly increase the time taken for outpatient appointments and has the potential to improve patient care and allow the department to accurately report outcomes.

Further development and liaison with the IT department is now recommended to overcome the identified limitations and integrate the use of such a database into routine clinical practice.

### 3.3. Introduction of Operative Note Templates

The staff involved in the pilot study found the use of a template to be safer, especially the recovery nurses who found the consistent order of the notes easier to follow than hand-written notes. They found that the tick box sections and reduction in writing made the template more legible and therefore they felt safer administering the postoperative care required. The surgical team did not rate the original template as highly as the nursing staff but found the second template quicker to fill out, safer, and more comprehensive.

The surgical team found the original FESS template unclear, and some disliked the illustrations. The surgeons found the modified form incorporating their feedback to be quicker, safer, and preferable to writing out their operative notes. They preferred the use of colour to stratify sections. They preferred to use their own drawings to illustrate intraoperative findings. The surgeons found the modified template to be quicker than hand-written notes. The sample of four templates showed complete, comprehensive notes which follow the RCS guidelines.

Following the introduction of the operative note template, we intend to compare hand-written operative notes with the operative note template for completeness when a sufficient number of templates have been used.

### 3.4. Construction of Electronic Discharge Summary Template

All discharges had adequate information but there was noncompliance with the RCP guidance; highlights included the following:

- 12 of the 15 eDSs were available for analysis;
- 7 out of 12 contained inconsistencies when compared to patient notes;
- half of all eDSs assessed contained “incomplete information” when compared to RCP guidance (see Table 1 for summary of detail);
- clinicians took a mean time of 5 minutes 25 seconds completing each electronic discharge summary.

This audit highlighted that there was room for improvement in the content quality of eDSs in rhinology. As a result custom “prompting” templates were constructed for FESS and septoplasty/turbinate surgery with a view to improving these parameters, improving patient safety, and saving time and money. These templates where constructed with two ideas in mind as follows.

(1) Addition of prewritten “delete as appropriate suggestions” for each subheading to aid clinicians to speed up completion of forms (see the following list).
   Please delete as appropriate suggestions:
  - chronic sinusitis refractory to medical treatment,
  - recurrent sinusitis,
  - nasal polyposis,
  - antrochoanal polyps,
  - sinus mucoceles,
  - excision of tumour,
  - cerebrospinal fluid (CSF) leak closure,
  - orbital decompression,
  - optic nerve decompression,
  - dacryocystorhinostomy (DCR),
  - choanal atresia repair,
  - foreign body removal,
  - epistaxis control.
(2) “Prompting words” to remind clinicians regarding certain content that had previously been omitted. See the following list for sample of new template:
   please include a brief clinical narrative and summary of advice for patients' GP, including medication recommendations,
   please include any RISKS and WARNINGS,
   what information has been given to patient regarding procedure?
   did the patient have the mental capacity to consent to this procedure? Y/N.

Following the introduction of these templates, a pilot study was conducted on patients undergoing septoplasty and turbinate surgery. An eDS was observed for completion time. The summary was then compared against RCP guidance using the same methodology as the initial audit.

Despite having a small sample, the audit of this pilot showed a 1-minute improvement in speed of completion of eDS (mean time taken was 4 minutes and 30 seconds). Prompting words improved adherence to RCP recommendations. A proposal has been put forward with a view to creating specific EDST's for common procedures across ENT.

## 4. Conclusion

Student-led interventions in specialist units can improve the quality of care given to patients. These four projects have shown the potential to ensure the delivery of safe, patient-centred healthcare that is both efficient and equitable and offer examples for other national units to consider in their practice.

## Figures and Tables

**Table 1:** 

Information subheading	% of records containing complete information
GP details	100%
Patient details	65.47%
Admission details	100%
Discharge details	75%
Clinical information	46.70%
Advice, recommendations, and future plan	55.55%
Person completing summary	100%

## References

[1] Gould B. E., Grey M. R., Huntington C. G. (2002). Improving patient care outcomes by teaching quality improvement to medical students in community-based practices. *Academic Medicine*.

[2] The National Institute for Health Research (NIHR) Collaboration for Leadership in Applied Health Research and Care (CLAHRC) South West Peninsula Telecon-Thyroid: are telephone consultations safe and effective in the management of thyroid disease?. http://clahrc-peninsula.nihr.ac.uk/research/telecon-thyroid.

[3] McVay M. R., Kelley K. R., Mathews D. L., Jackson R. J., Kokoska E. R., Smith S. D. (2008). Postoperative follow-up: is a phone call enough?. *Journal of Pediatric Surgery*.

[4] Gray R. T., Sut M. K., Badger S. A., Harvey C. F. (2010). Post-operative telephone review is cost-effective and acceptable to patients. *Ulster Medical Journal*.

[5] Marshall S., Haywood K., Fitzpatrick R. (2006). Impact of patient-reported outcome measures on routine practice: a structured review. *Journal of Evaluation in Clinical Practice*.

[6] Kapoor K., Hopkins C. (2015). Underutilisation of the British Rhinological Society minimum electronic dataset in an age of mandatory reporting; an investigation. *Clinical Otolaryngology*.

[7] Ghani Y., Thakrar R., Kosuge D., Bates P. (2014). ‘Smart’ electronic operation notes in surgery: an innovative way to improve patient care. *International Journal of Surgery*.

[8] Moore L., Churley-Strom R., Singal B., O'Leary S. (2009). Laparotomy operative note template constructed through a modified Delphi method. *American Journal of Obstetrics & Gynecology*.

[9] Cowan D. A., Sands M. B., Rabizadeh S. M. (2007). Electronic templates versus dictation for the completion of Mohs micrographic surgery operative notes. *Dermatologic Surgery*.

[10] Moegan D., Fisher N., Ahmad A., Alam F. (2009). Improving operation notes to meet British orthopaedic association guidelines. *Annals of the Royal College of Surgeons of England*.

[11] Barritt A. W., Clark L., Cohen A. M., Hosangadi-Jayedev N., Gibb P. A. (2010). Improving the quality of procedure-specific operation reports in orthopaedic surgery. *Annals of the Royal College of Surgeons of England*.

[12] Bateman N. D., Carney A. S., Gibbin K. P. (1999). An audit of the quality of operation notes in an otolaryngology unit. *Journal of the Royal College of Surgeons of Edinburgh*.

[13] GMC (2011). *Good Medical Practice*.

[14] The Royal College of Surgeons of England (2010). *Good Surgical Practice*.

[15] Hoffer D. N., Finelli A., Chow R. (2012). Structured electronic operative reporting: comparison with dictation in kidney cancer surgery. *International Journal of Medical Informatics*.

[16] O'Leary K. J., Liebovitz D. M., Feinglass J. (2009). Creating a better discharge summary: improvement in quality and timeliness using an electronic discharge summary. *Journal of Hospital Medicine*.

[17] O'Leary K. J., Liebovitz D. M., Feinglass J., Liss D. T., Baker D. W. (2006). Outpatient physicians' satisfaction with discharge summaries and perceived need for an electronic discharge summary. *Journal of Hospital Medicine*.

[18] HSCIC (2010). *Electronic 24-Hour Discharge Summary Implementation*.

[19] Horwitz L. I., Jenq G. Y., Brewster U. C. (2013). Comprehensive quality of discharge summaries at an academic medical center. *Journal of Hospital Medicine*.

[20] Mamo J. P. (2014). Electronic discharge summaries—are they being done and do they have the required information?. *Irish Medical Journal*.

[21] Hammad E. A., Wright D. J., Walton C., Nunney I., Bhattacharya D. (2014). Adherence to UK national guidance for discharge information: an audit in primary care. *British Journal of Clinical Pharmacology*.

[22] Kripalani S., LeFevre F., Phillips C. O., Williams M. V., Basaviah P., Baker D. W. (2007). Deficits in communication and information transfer between hospital-based and primary care physicians: implications for patient safety and continuity of care. *Journal of the American Medical Association*.

[23] Academy of Medical Royal Colleges (2013). *Standards for the Clinical Structure and Content of Patient Records*.

[24] Maurice A. P., Chan S., Pollard C. W. (2014). Improving the quality of hospital discharge summaries utilising an electronic prompting system. *BMJ Quality Improvement Reports*.

[25] Ladds E., Betteridge F., Yamamoto S., Gupta-Jessop T. (2015). Improving the quality of discharge summaries for elective surgical procedures at North Bristol NHS Trust. *BMJ Quality Improvement Reports*.

